# The development of an objective method for evaluating transient sleeping environments

**DOI:** 10.1186/2046-7648-4-S1-A141

**Published:** 2015-09-14

**Authors:** Olga Troynikov, Nazia Nawaz, Christopher Watson

**Affiliations:** 1 Human Ecology and Clothing Science Research Group, School of Fashion and Textiles, RMIT University, Melbourne, Australia

## Introduction

Poor sleep is the key sleep attribute that affects the overall recuperative quality of the sleep state [1]. A common approach in human sleep testing is to use subjects in an environmental chamber [2-4]. One of the significant limitations of this testing is its subjectivity and the small number of subjects [5]. The use of thermal manikins to measure thermal and vapour resistance is a standard method adopted for assessment of sleeping bags and has also been used to measure these attributes of a range of bedding systems, as well as different sleepwear [6].

## Methods

A compacted transient experimental protocol totalling 3 hours 20 minutes was established in order to determine the possible impact of the different bedding systems and mattresses on sleeping bedding microclimates, using a 20 zone sweating thermal manikin, Newton. A dynamic experimental cycle of different heating and sweating phases was used to simulate the sleeping human metabolic and sweating activity conditions through different sleep phases. The manikin was placed on the experimental mattress, either latex or inner spring, in the experimental bedding systems in a controlled environmental chamber. Temperature and humidity in the next to skin microenvironment were dynamically measured at different sites of the manikin's body by using temperature and humidity sensor arrays. The environmental temperature was set at 17 °C, and a relative humidity rh was set at 50 %. All individual elements of the sleeping systems were conditioned prior to each experiment in the climatic chamber at the relevant test conditions of 17 °C and rh of 50 % for 12 hours.

## Results

The study found that the latex mattress ensemble exhibits a higher overall bedding micro environmental temperature than the inner spring mattress ensemble with all other variables being constant. This resulted in up to 3 °C higher micro environmental temperature for all bedding systems by the end of the experiment. Furthermore, the micro environmental temperature differences between the front and the back zones of the manikin for the latex mattress reach 2.5 °C. The latex mattress ensemble also exhibits higher overall microclimate humidity levels in comparison to the inner spring mattress ensemble, with all other variables being constant, with humidity levels for the back zones being double for the latex ensemble in comparison to the inner spring ensemble and reaching 50 % RH. In particular, the humidity levels for the microclimate at the back zones of the manikin are significantly higher for the latex system in comparison to the inner spring mattress system. Both Independent sample t-test of experimental data and Paired Samples Statistical analyses of data produced p-values less than 0.05. This allowed the conclusion that there is a significant difference between micro environments produced by the experimental bedding systems and mattress ensembles.

## Conclusion

The developed objective method and exploratory initial experimental results of this study suggest that it is possible to objectively identify bedding systems which provide moderate micro environmental temperature fluctuations and steady humidity profiles during the sleep cycle.
